# Characterization of a *Trichinella spiralis* putative serine protease. Study of its potential as sero-diagnostic tool

**DOI:** 10.1371/journal.pntd.0006485

**Published:** 2018-05-14

**Authors:** Ge Ge Sun, Yan Yan Song, Peng Jiang, Hua Na Ren, Shu Wei Yan, Yue Han, Ruo Dan Liu, Xi Zhang, Zhong Quan Wang, Jing Cui

**Affiliations:** Department of Parasitology, Medical College, Zhengzhou University, Zhengzhou, P. R. China; Istituto Superiore di Sanità, UNITED STATES

## Abstract

**Background:**

Trichinellosis is a serious zoonositc parasitosis worldwide. Because its clinical manifestations aren’t specific, the diagnosis of trichinellosis is not easy to be made. *Trichinella spiralis* muscle larva (ML) excretory–secretory (ES) antigens are the most widely applied diagnostic antigens for human trichinellosis, but the major drawback of the ES antigens for assaying anti-*Trichinella* antibodies is the false negative in the early *Trichinella* infection period. The aim of this study was to characterize the *T*. *spiralis* putative serine protease (TsSP) and to investigate its potential use for diagnosis of trichinellosis.

**Methodology/Principal findings:**

The full-length TsSP sequence was cloned and expressed, and recombinant TsSP (rTsSP) was purified by Ni-NTA-Sefinose Column. On Western blotting analysis the rTsSP was recognized by *T*. *spiralis*-infected mouse serum, and the natural TsSP was identified in *T*. *spiralis* ML crude and ES antigens by using anti-rTsSP serum. Expression of TsSP was detected at various *T*. *spiralis* developmental stages (newborn larvae, muscle larvae, intestinal infective larvae and adult worms). Immunolocalization identified the TsSP principally in cuticles and stichosomes of the nematode. The sensitivity of rTsSP-ELISA and ES-ELISA was 98.11% (52/53) and 88.68% (47/53) respectively (*P* > 0.05) when the sera from trichinellosis patients were examined. However, while twenty-one serum samples of trichinellosis patients’ sera at 19 days post-infection (dpi) were tested, the sensitivity (95.24%) of rTsSP-ELISA was distinctly higher than 71.43% of ES-ELISA (*P* < 0.05). The specificity (99.53%) of rTsSP-ELISA was remarkably higher than 91.98% of ES-ELISA (*P* < 0.01). Only one out of 20 serum samples of cysticercosis patients cross-reacted with the rTsSP. Specific anti-*Trichinella* IgG in infected mice was first detected by rTsSP-ELISA as soon as 7 dpi and antibody positive rate reached 100% on 10 dpi, whereas the ES-ELISA did not permit detection of 100% of infected mice before 16 dpi.

**Conclusions:**

The rTsSP is a potential early diagnostic antigen for human trichinellosis.

## Author summary

Trichinellosis is an important parasitic zoonosis, and has a public health hazard and an economic impact on the safety of animal food. The diagnosis of trichinellosis is difficult and it is often misdiagnosed. There is an evident 2–3 week window stage between clinical manifestations and the anti-*Trichinella* IgG positive. Serine protease is a superfamily of proteolytic enzymes and exerts a major role in tissue invasion, larval development and survival of the parasites. A *T*. *spiralis* putative serine protease (TsSP) was characterized in ES proteins of *T*. *spiralis* intestinal infective larvae and adult worms by the immunoproteomics with early infection serum. In this study, the TsSP was expressed and purified. The results revealed that the TsSP was expressed at various *T*. *spiralis* stages (newborn larvae, muscle larvae, intestinal infective larvae and adult worms) and it was principally located in cuticle and stichosome of the nematode. The rTsSP was sensitive and specific for detection of anti-*Trichinella* IgG, and could be regarded as an early diagnostic marker of trichinellosis.

## Introduction

Trichinellosis is an important food-borne parasitic disease worldwide. *Trichinella* infection occurs by ingesting raw or undercooked meat containing *Trichinella* muscle larvae [1]. *T*. *spiralis* is the main etiological agent of trichinellosis [2]. Outbreak of human trichinellosis was recorded in 55 countries around the world, and there were 65,818 cases and 42 deaths from trichinellosis reported from 41 countries during 1986–2009 [3]. Fifteen outbreaks of trichinellosis were documented in mainland China during 2004–2009 and pork is the dominating infection source [4,5]. A survey showed that the prevalence of porcine *Trichinella* infection in small pig farms in central China varied from 0.61% to 3.79% during 2010–2015, although the larval burdens in infected pigs was less than 2 larvae per gram of muscles [6,7]. Hence, trichinellosis has a public health hazard and an economic impact in meat food safety [8].

Since the symptoms and signs of trichinellosis aren’t specific, the diagnosis of trichinellosis isn’t easy to be established according to the clinical manifestations of this disease [9]. At present, the serological test widely applied for diagnosis of human trichinellosis is to detect anti-*Trichinella* IgG by ELISA and Western blotting with *T*. *spiralis* muscle larvae (ML) excretory/secretory (ES) antigens [10], but the principal drawback is the false negative in the early phase of this infection [11]. The occurrence of a 2–3 week window period of anti-*Trichinella* antibody negative is probable duo to the fact that the major ML ES antigen epitopes are the phase-specific for ML and not recognized by anti-*Trichinella* antibodies triggered by intestinal infective larvae (IIL) at 6 hours post infection (hpi) and adult worm (AW) at 3 dpi of the nematode in the early stage of *Trichinella* infection [12]. The ES antigens generated by the IIL and AW might firstly be exposed to host’s immune system and induced the generation of specific antibodies against the nematode. The recent investigation indicated that AW crude antigen positively reacted with swine and mouse infection sera at 7–8 dpi [13,14]. On Western blot analysis, the recombinant *T*. *spiralis* cystatin-like protein (rTsCLP) of IIL stage was probed by porcine infection sera at 15–20 dpi [15]. Anti-*Trichinella* IgG in serum samples of *T*. *spiralis*-infected mice was detected by ELISA using ES antigens of AW or IIL as soon as 8 dpi [16,17]. Therefore, it is likely that the diagnostic markers for early *Trichinella* infection will be exploited from the enteral worms of *T*. *spiralis* [18].

In our previous studies, immunoproteomics was used to investigate the early antigens for serodiagnosis of trichinellosis, and a putative serine protease was identified in the ES proteins from *T*. *spiralis* IIL and AW by mouse infection sera at 8–10 dpi and early trichinellosis patients’ sera at 19 dpi [12,19]. Additionally, the *T*. *spiralis* putative serine protease (TsSP) (GenBank accession no. ABY60762) was highly expressed in surface proteins of IIL stage compared with those of ML stage [20]. The aim of this study was to characteriz the TsSP and investigate the prospective diagnostic values of recombinant TsSP (rTsSP) for early trichinellosis.

## Materials and methods

### Ethics statement

The present study was performed in the light of National Guidelines for Experimental Animal Welfare (MOST of People’s Republic of China, 2006). All animal care and use in our research were reviewed and approved by the Life Science Ethics Committee of Zhengzhou University (No. SCXK 2015–0005). All the human serum samples were collected from adults, and the written informed consent was acquired from the adults before samples were used.

### Parasites and experimental animals

*T*. *spiralis* isolate (ISS534) utilized in our study was acquired from a naturally infected domestic swine in Henan Province of central China. This isolate was passaged in BALB/c mice in our department. Six-week-old female BALB/c mice were provided by the Experimental Animal Center of Zhengzhou University (Zhengzhou, China). Mice were kept with specific pathogen-free conditions under suitable temperature and humidity.

### Serum samples

Fifty-three serum samples from trichinellosis patients were obtained from two outbreaks of human trichinellosis in southwestern China [17]. The sera from patients with paragonimiasis (n = 20), schistosomiasis (n = 34), clonorchiasis (n = 7), cysticercosis (n = 20), echinococcosis (n = 20) and sparganosis (n = 7) were conserved in our laboratory. The diagnosis of these patients was established by fecal parasitological examination or serum specific antibody detection [11,21]. The sera from 104 presumably healthy persons, who came from non-endemic areas of trichinellosis and assayed negative for the before-mentioned helminthiases, were also examined in our study.

In order to observe the dynamics of anti-*Trichinella* IgG, nine mice were infected orally with 300 *T*. *spiralis* ML. About 100 μl of tail blood was collected from infected mice on alternate days during 2–30 dpi and serums were isolated. Serum samples from normal mice were obtained and utilized as the negative control.

### Worm collection and antigen preparation

*T*. *spiralis* ML were obtained from experimentally infected mice at 35 dpi by using the artificial digestion method as described [22,23]. The IIL were recovered from intestines of the infected mice at 6 hpi [24], and the adult worms (AW) were separated from mouse duodenum and jejunum at 3 and 6 dpi, respectively [17]. The newborn larvae (NBL) were obtained from the adult females cultured in vitro in RPMI-1640 with 10% fetal bovine serum (FBS; Gibco) at 37°C in 5% CO_2_ for 24 h [25]. The crude soluble antigens of AW, NBL, ML and IIL, and the ML ES antigens were produced as described [26,27].

### Sequence analysis of TsSP gene

The complete TsSP cDNA sequence was acquired from the GenBank database with accession no. ABY60762. The Pepstats software was applied to predict molecular weight (MW), isoelectric point (pI) and transmembrane helices of the TsSP protein [28,29]. The putative N-glycosylation site was verified with the NetNGly1.0 server (http://www.cbs.dtu.dk/services/NetNGlyc/). The potential B and T cell epitope of the TsSP was calculated with the DNAStar software and the online server of BepiPred (http://www.cbs.dtu.dk/services/BepiPred/), respectively [30]. The tertiary structure of the TsSP protein was predicted on the Expasy website (http://web.expasy.org/). The identification of protein motifs and catalytic triad of the TsSP was from aligning the multiple protein sequences [31].

### Cloning, expression, and purification of rTsSP

The total ML RNA was extracted with Trizol reagent (Invitrogen, USA). The full-length TsSP sequences were amplified via PCR with specific primers carrying enzyme BamHI and PstI sites (bold and italicized) (5'-G***GGATCC***ATGATCCTTTTCAAGTGCTTATTTCT-3' and 5'-GCG***CTGCAG***TCAGCAAACTCAATTTATTTAGAT-3'). The TsSP gene coding regions without a 18 amino acid signal peptide were produced by PCR with oligonucleotide primers carrying enzyme BamHI and PstI sites (bold and italicized) (5'-TTC***GGATCC***AATTATGAA TGTGGCACCTTAC-3' and 5'-CCG***CTGCAG***TTAACGGAAAAAAGTGAATGAT-3'). PCR amplification reaction included 25μl premix (DNA polymerase, dNTPs and PCR buffer), 0.5 μl cDNA, 0.4μl DNA polymerase, 1.0 μl 10 μM of each primer, 22 μl ddH_2_O. The cycling procedure was as follows: 98°C for 5 min; 30 cycles of at 94°C for 3min, 94°C for 45 s,60°C for 45 s, 72°C for 90 s, and finally 5 min at 72°C. The final purified PCR product was digested and cloned into the pGEM-T vector (Promega, USA), then sub-cloned into the pQE-80L carrying the N-terminus His-tag (Novagen, USA). The recombinant pQE-80L/TsSP was transformed into *Escherichia coli* BL21 (DE3) (Novagen). The rTsSP expression was induced by using 0.5 mM IPTG for 4 h at 30°C. The rTsSP were purified with a Ni-NTA His-tag affinity kit (Novagen). The rTsSP protein were identified on SDS–PAGE analysis [32]. The concentration of the rTsSP protein was assayed as described [33].

### Phylogenetic analysis of the TsSP

The sequences of serine protease homologues from other organisms were aligned using the default settings in the program Clustal X [34]. The phylogenetic relationship among TsSP and other homologues was assayed by using a phylogenetic tree constructed in the MEGA 5.0 under the maximum parsimony algorithm with 1 000 bootstrap replications [35].

### Preparation of anti-rTsSP serum

Thirteen BALB/c mice were immunized with rTsSP. Each mouse was injected abdomen subcutaneously with 20 μg of rTsSP emulsified in Freund’s complete adjuvant, then the mice were boosted twice with the same amount of rTsSP emulsified with Freund’s incomplete adjuvant at an intervals of 10 days [36]. About 50 μl of blood sample from immunized mice were obtained at 10 days after final immunization and serum anti-rTsSP antibody titer was assayed by ELISA with 2 μg/ml rTsSP as coating antigen [37].

### Western blotting analysis

Samples consisted of 5μg rTsSP, 15μg ML crude antigens and ML ES antigens per lane. The protein was separated on SDS-PAGE with 12% separation gel, subsequently transferred onto the membranes (Millipore, USA) at 18 V for 35 min in a semi-dry transfer cell (Bio-Rad, USA) [26]. The membrane was blocked by 5% skim milk in Tris–buffered saline with 0.05% Tween-20 (TBST) at room temperature for 2 h, and incubated with 1:100 dilutions of different sera (anti-rTsSP serum, serum of *T*. *spiralis-*infected mice collected at 42 dpi, immune serum from mice immunized with ML ES and crude antigens, and uninfected normal mouse serum) at 4°C overnight. Following being washed, the membrane was incubated with 1:10 000 dilutions of HRP-conjugated goat anti-mouse IgG at 37°C for 1 h. The membrane was colored by use of 3,3’-diaminobenzidine tetrahydrochloride (DAB; Sigma), and terminated by washing the membrane with deionized water.

To detect the relative TsSP expression level in *T*. *spiralis* different stages, 15 μg/lane of soluble proteins of ML, IIL, AW and NBL was separated with SDS-PAGE and identified by Western blotting with 1:100 dilutions of anti-rTsSP serum [38]. Rabbit anti-β-actin antibody diluted at 1:400 was utilized as a quantitative protein control to detect β-actin expression. After it was washed three times with TBST, the color development was performed by the enhanced chemiluminescence (ECL) kit (CWBIO, Beijing, China) [39]. The relative expression level of the TsSP protein at various *T*. *spiralis* phases was determined with Image J software.

### RT-PCR assay of TsSP transcription

Total RNA was extracted respectively from diverse *T*. *spiralis* phases (ML, IIL, AW, and NBL) with Trizol reagent (Invitrogen). The RT-PCR was carried out according to the previous report [38]. By using as an internal control, *T*. *spiralis* glyceraldehyde-3-phosphate dehydrogenase (GAPDH, GenBank accession No. AF452239) was amplified as a housekeeping gene in our study. PBS was used as a negative control template in all PCR assays.

### ELISA

The crude proteins from different *T*. *spiralis* phases (NBL, ML, IIL and AW) and ES proteins from AW, ML and IIL were prepared as described [27,40]. The above-mentioned antigens and rTsSP were diluted to a final concentration of 1.5 μg/ml. The ELISA procedure was performed as described previously [11]. Briefly, the microtiter plate was coated with the antigens at 4°C overnight. Following being washed with PBST, it was blocked with 5% skimmed milk in PBST at 37°C for 2 h. After washing again, the plate was incubated at 37°C for 1 h with 1:200 dilutions of trichinellosis patients’ serum or 1:100 dilutions of mouse serum, subsequently incubated with HRP-conjugated anti-human/mouse antibody IgG (1:10 000) at 37°C for 1h. After the last washing, the coloration was developed by incubation with o-phenylenediamine dihydrochloride (OPD; Sigma) plus 30% H_2_O_2_ for 30 min. The reaction was ceased by 2M H_2_SO_4_. The absorbance (optical density, OD) was measured at 490 nm, and all serum samples were assayed in duplicate. The ratio < 2.1 of assayed serum/negative serum OD values was taken as negative and the ratio ≥2.1 as positive [41]. The cut-off value of rTsSP-ELISA and ES-ELISA for detection of the patient’s serum was 0.35 and 0.45, respectively. The cut-off value of the above two ELISA for detection of experimentally infected mice was 0.20 and 0.21, respectively.

### Immunofluorescent test (IFT)

To confirm whether the TsSP expressed on the surface of *T*. *spiralis* diverse stages, the whole worms were used in IFT [42]. Additionally, the tissue sections with 3 μm thickness of female adults at 3 dpi, ML and IIL were separately cut by a microtome. The intact nematodes and their sections were blocked in 5% normal goat serum diluted with PBS, and incubated using a 1:10 dilution of mouse immune serum, infection serum or negative control serum. FITC-labeled goat anti-mouse IgG diluted at 1:100 (Santa Cruz, USA) was utilized as the second antibody. After they were washed with PBST, the intact nematode and sections were examined under a fluorescent microscopy (Olympus, Japan).

### Statistical analysis

The statistical analysis of data was carried out by using SPSS 17.0 software. All the data were shown as arithmetic means ± standard deviation (SD). The comparison of the TsSP expression level in *T*. *spiralis* various stages was performed with one-way ANOVA. Chi-square test was used to determine the difference between groups. The statistical test was regarded significant at *P* < 0.05.

## Results

### Bioinformatic analysis of TsSP sequences

**Fig 1:**
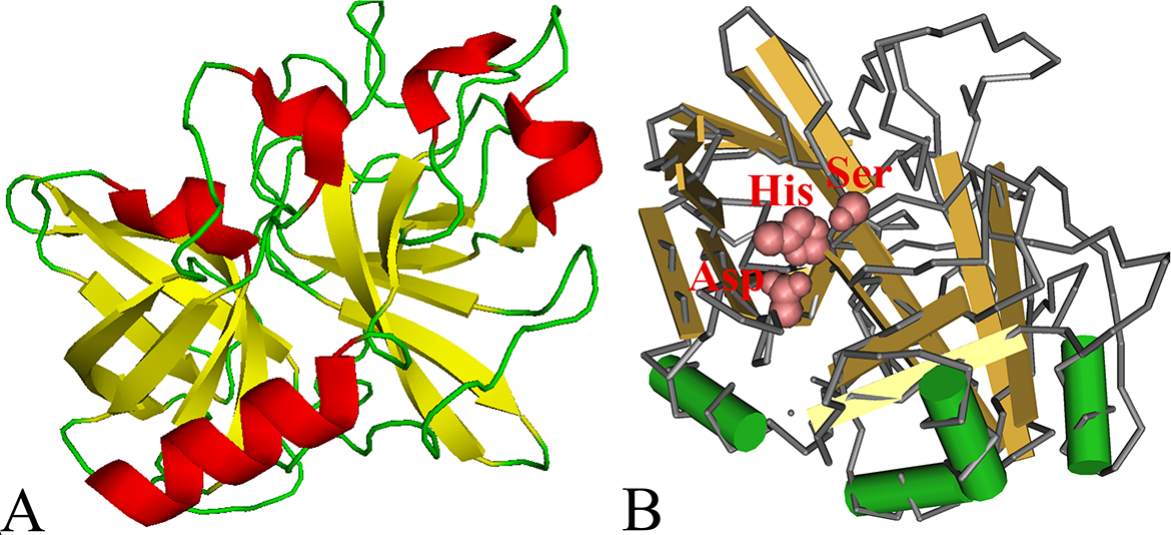
The predicted three-dimensional model of *T*. *spiralis* TsSP protein. A: The predicted three-dimensional structure of TsSP protein, there are 4 α-helixes (in red), 14 β-strand (in yellow), and 19 irregular coils (in green); B: Catalytic residues Ser-His-Asp form a pocket-shaped functional domain. The active site of TsSP was highlighted with red color.

Bioinformatics analysis revealed that the full-length cDNA sequence of the TsSP gene was 1372 bp (CDS: 2–1290 bp). The predicted MW and pI of TsSP were 47.55 kDa and 8.73, respectively. The signal peptides were located at 1–18 aa (MILFKCLFLLAYTTLAFA). The mature serine protease consisted of 411 amino acid residues of 45.2 kDa, and no transmembrane helix was detected, indicating that the TsSP is a secretory protein. Only one N-glycosylation site 78–81 (NGSQ) of the TsSP was identified. Secondary structures of the TsSP had 18 potential B cell epitopes. The SMART analysis results demonstrated that the TsSP had a domain (at 37-277aa) of trypsin-like serine protease carrying an active site of classic catalytic triad. In three-dimensional model, the motif of catalytic triad (Serine–Histidine–Aspartate) constituted a functional domain carrying substrate binding sites (Fig 1).

### Sequence alignment and the comparison of TsSP

**Fig 2:**
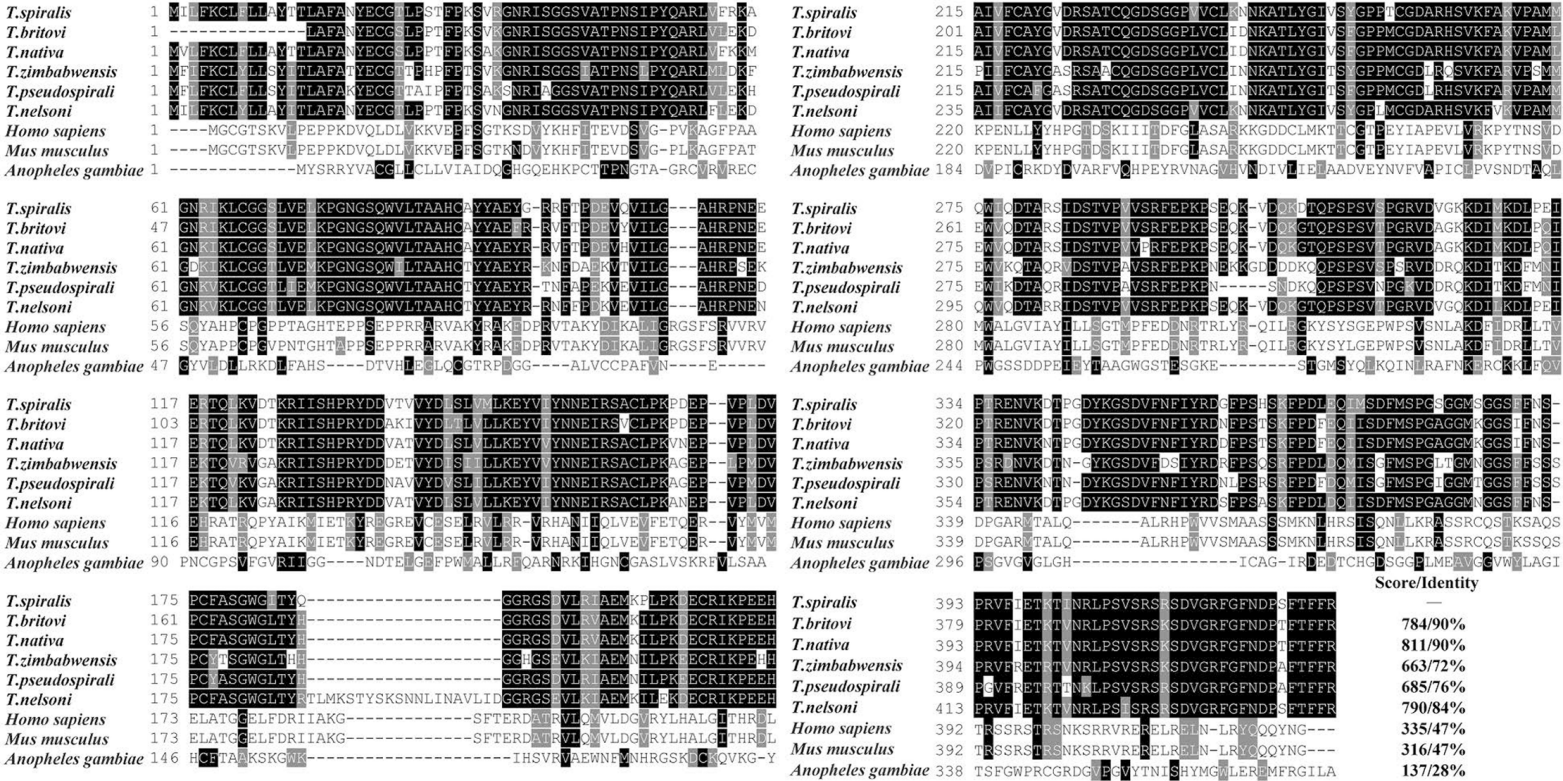
Sequence alignment of serine protease from *Trichinella spiralis* (ABY60762) with other species of the genus *Trichinella* and other organisms. The serine proteases was from *Trichinella* spp.: *T*. *britovi* (KRY59723.1), *T*. *nativa* (KRZ48330.1), *T*. *zimbabwensis* (KRZ02345.1), *T*. *pseudospirali* (KRY01512.1), *T*. *nelsoni* (KRX27556.1), *Homo sapiens* (CAB91984.1), *Mus musculus* (EDL11329.1), *Anopheles gambiae* (CAB90819.1). The multiple sequences alignment was performed in the Clustal X and displayed using BOXSHADE. Black shade indicated that residues identical to TsSP, and conservative substitutions were shaded grey. The numbers at the end of each sequence represent the score and percent identity to TsSP.

**Fig 3:**
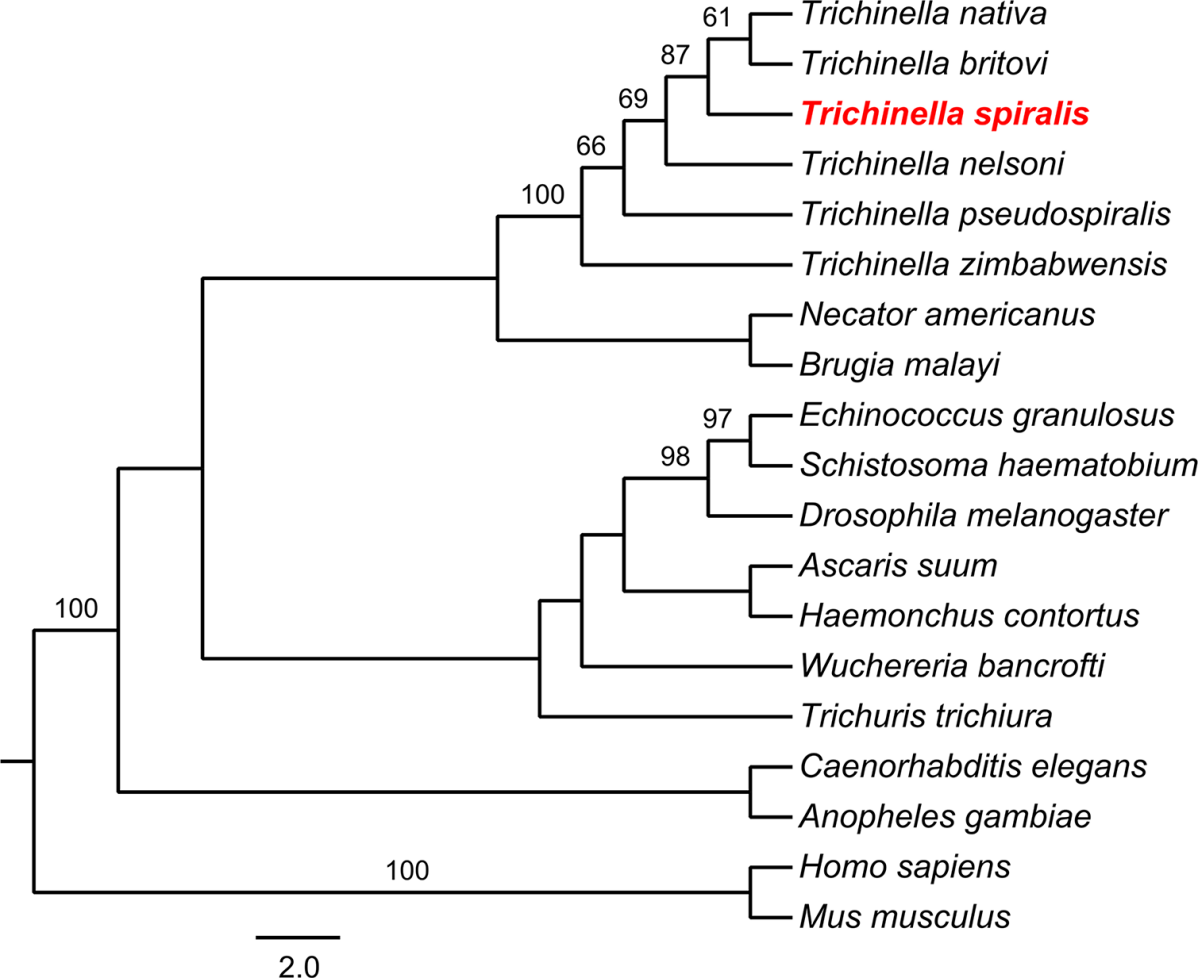
Cladogram of analysis of TsSP. The maximum parsimony tree of serine protease from 19 organisms calculated using MEGA. The GenBank accession numbers of each serine protease are as follows: *Trichinella spiralis* (ABY60762.1), *Trichinella nativa* (KRZ48330.1), *Trichinella nelsoni* (KRX27556.1), *Trichinella britovi* (KRY59723.1), *Trichinella pseudospiralis* (KRY01512.1), *Trichinella zimbabwensis* (KRZ02345.1), *Homo sapiens* (CAB91984.1), *Mus musculus* (EDL11329.1), *Caenorhabditis elegans* (CCD65324.1), *Drosophila melanogaster* (NP_001262565.1), *Trichuris trichiura* (CDW53654.1), *Ascaris suum* (ERG81033.1), *Necator americanus* (XP_013308358.1), *Brugia malayi* (XP_001900088.1), *Wuchereria bancrofti* (EJW79555.1), *Haemonchus contortus* (CDJ82043.1), *Echinococcus granulosus* (EUB62466.1), *Anopheles gambiae* (CAB90819.1), *Schistosoma haematobium* (KGB34910.1). Bootstrap values which are higher than 80 are indicated on branches.

A homology comparison of TsSP and other serine protease orthologues in the genus *Trichinella* was determined (Fig 2), among these sequences, the highest homology was between *T*. *spiralis* and *T*. *nativa* (with 90% identity). As shown in the phylogenetic tree generated with TsSP and its orthologues (Fig 3), the *Trichinella* genus was displayed as a monophyletic group with bootstrap value of 87. Within the *Trichinella*, the close relationships among *T*. *spiralis*, *T*. *nativa* and *T*. *britovi* were supported with a high bootstrap value (95).

### Cloning and expression of recombinant TsSP

**Fig 4:**
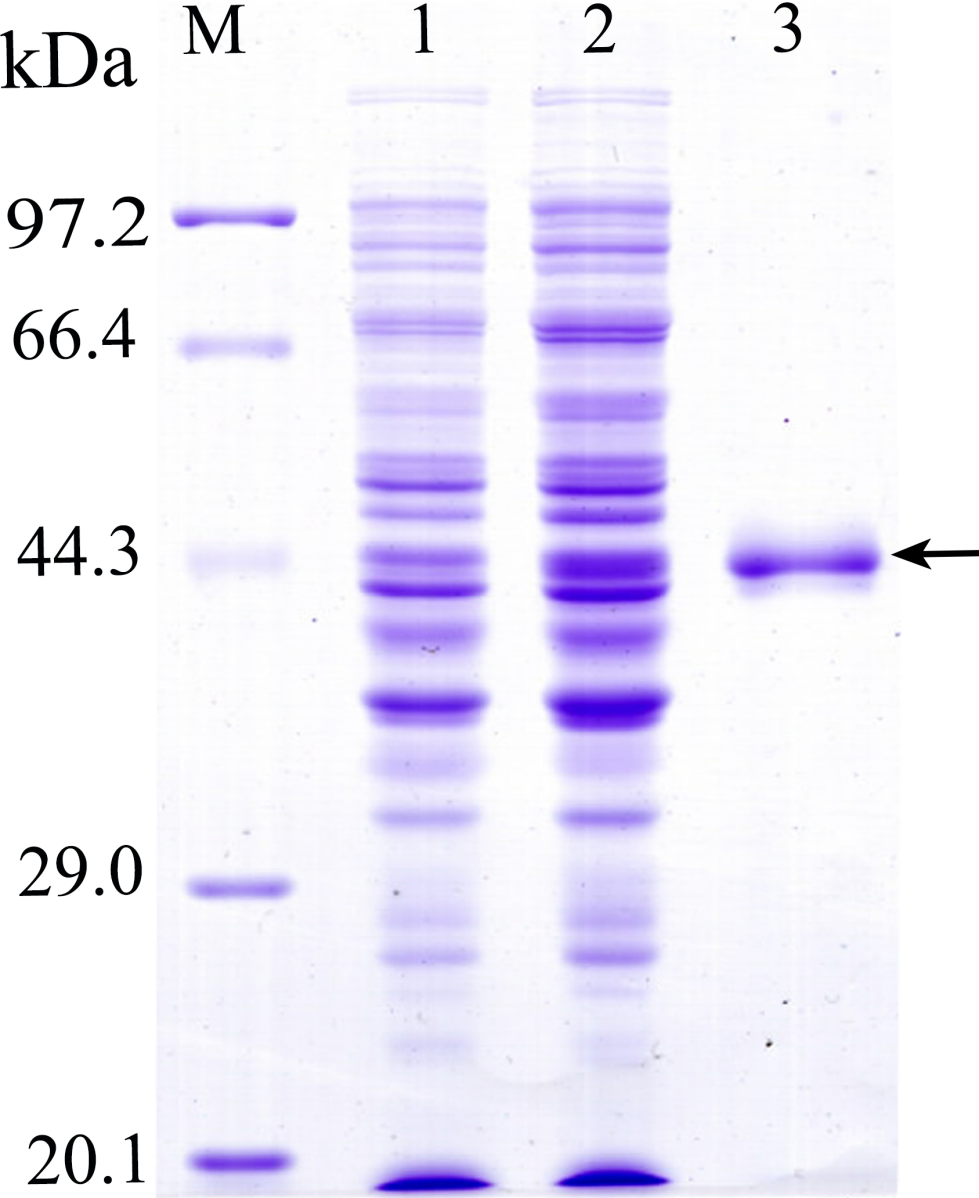
SDS-PAGE analysis of the rTsSP. M: protein molecular weight marker; 1: protein lysates from recombinant *E*. *coli* before being induced, 2: protein lysates of recombinant *E*. *coli* induced by IPTG; 3: rTsSP purified by Ni-NTA-Sefinose Column. Arrow indicates rTsSP.

The complete TsSP cDNA sequences without signal peptide were 1236 bp. The open reading frame (ORF) of TsSP encoded a 45.2 kDa protein of 411 amino acids. The TsSP coding sequences were cloned into the pQE-80L. Following induction, SDS-PAGE analysis showed that the recombinant bacteria harboring pQE-80L/TsSP expressed a protein band with 45.2 kDa. After being purified, the rTsSP had a single distinct protein band (Fig 4). The molecular weight (45.2 kDa) of the rTsSP was consistent with its expected size.

### Humoral immune responses elicited by immunization with rTsSP

**Fig 5:**
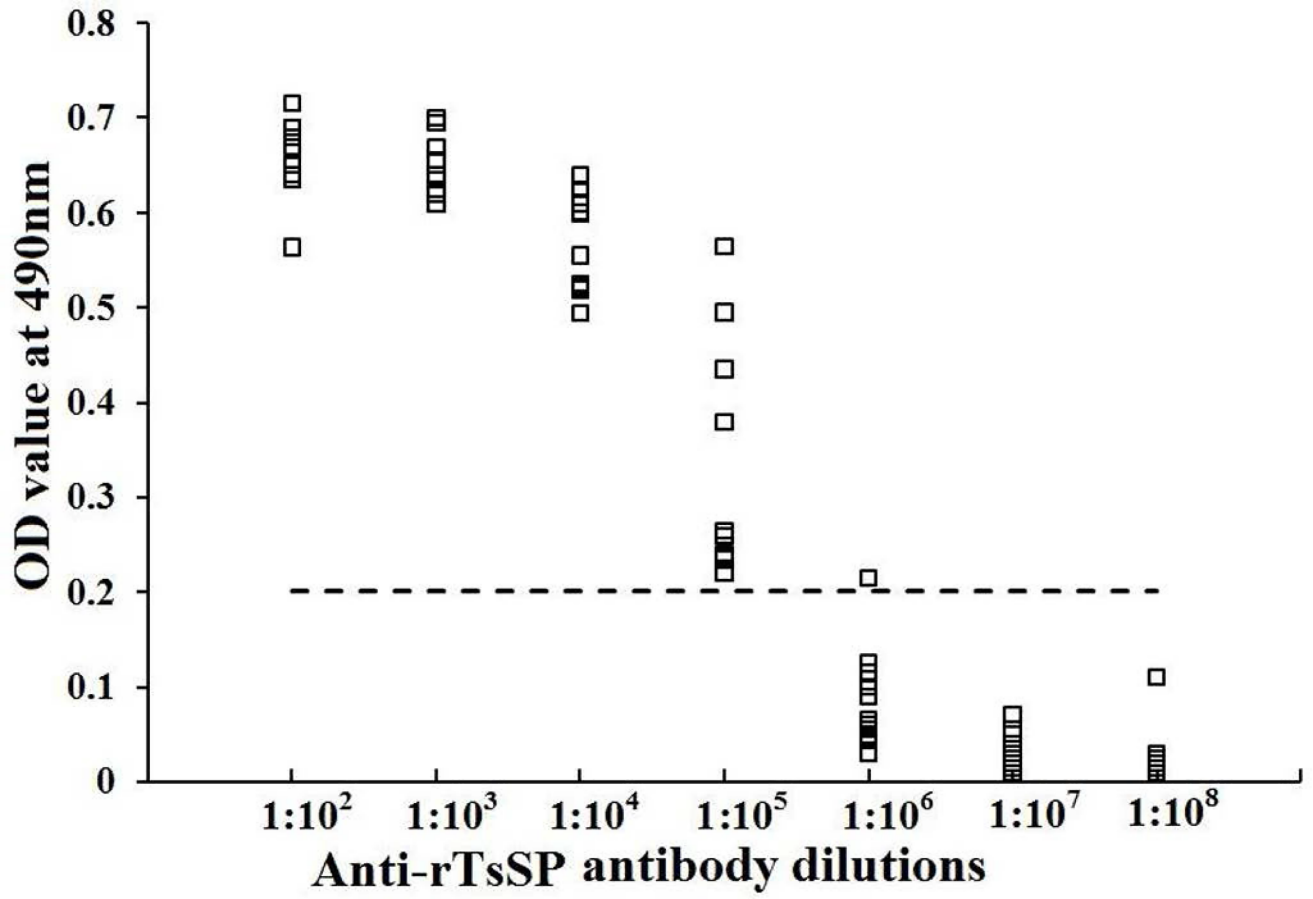
Serum anti-rTsSP IgG titers determined by ELISA. Microtiter plates were coated with 2 μg rTsSP /ml and incubated overnight at 4°C. After being blocked with 5% nonfat milk, the plates were incubated at 37°C for 1 h with different dilutions of immune sera. Normal mouse sera (n = 20) diluted at 1:100 were assayed as negative controls. HRP-conjugated IgG was utilized as the second antibodies. The coloration was developed by incubation with the substrate OPD. The absorbance at 490 nm was assayed following adding 2 M H_2_SO_4_. The cut-off values (0.202) are expressed with a dotted line.

To determine humoral immune responses to rTsSP in immunized mice, serum specific anti-rTsSP IgG titers at days 10 after the final immunization were measured by ELISA. As shown in Fig 5, anti-rTsSP antibodies could be triggered by the immunization with rTsSP. The titer of serum anti-rTsSP IgG was 1:10^5^ following the last immunization, indicating that the rTsSP has a high immunogenicity.

### Western blot analysis of the rTsSP

**Fig 6:**
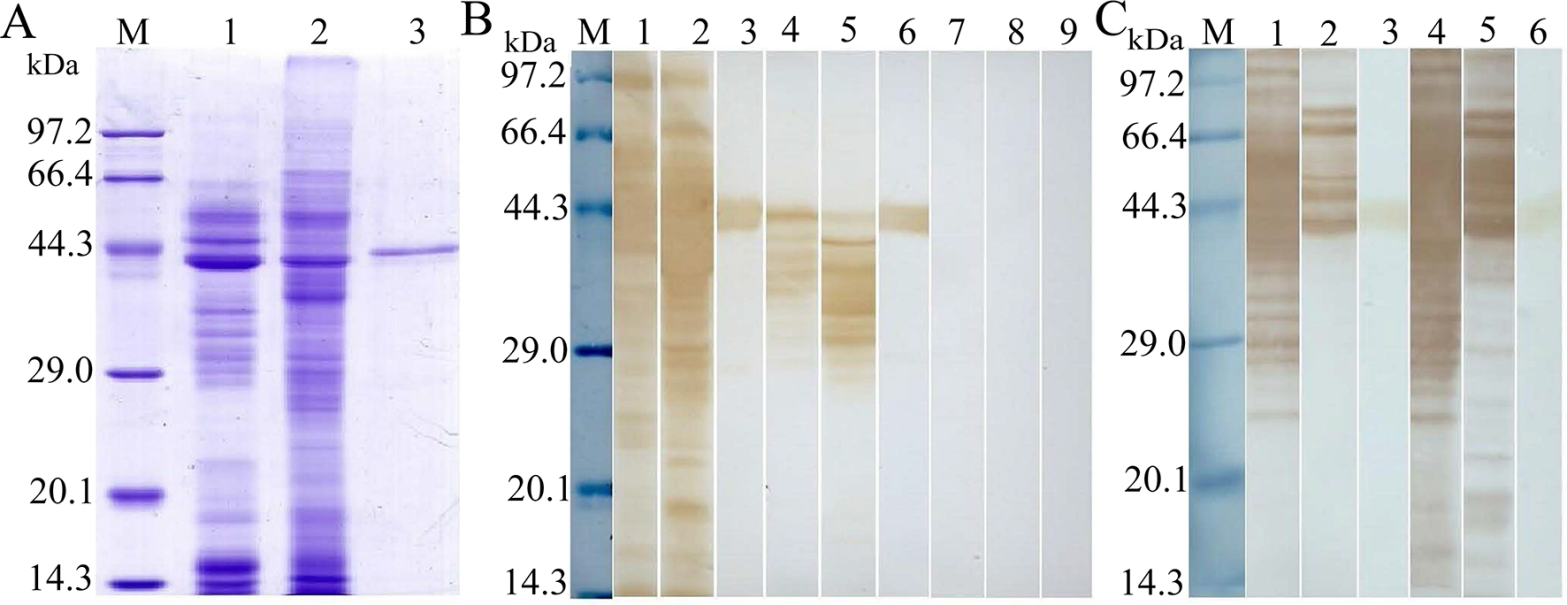
Western blot analysis of rTsSP antigenicity. **(A)** SDS-PAGE analysis of ML ES antigens from *T*. *spiralis* (lane 1), ML crude antigen (lane 2), and rTsSP (lane 3). **(B)** Western blotting of the rTsSP. *T*. *spiralis* ML ES antigens (lane 1) and ML crude antigens (lane 2) and rTsSP (lane 3) were probed with mouse infection sera. The natural TsSP protein in ML ES (lane 4), ML crude antigens (lane 5) and rTsSP (lane 6) were identified by using anti-rTsSP serum. Normal mouse sera did not probe the ES (lane7) and crude antigens (lane 8), and rTsSP (lane 9). (**C**) Western blotting of ML ES (lane 1 and 4) and crude antigens (lane 2 and 5), and rTsSP (lane 3 and 6) probed with immune sera from mice vaccinated with ES and crude antigens.

The results of SDS-PAGE analysis showed that the ML crude antigens had 44 bands with MW of 14.7–97.2 kDa, ML ES antigens had 29 bands with 14.4–96.3 kDa, and the rTsSP had only one band with 45.2 kDa (Fig 6A). On Western blot analysis the rTsSP was probed with anti-rTsSP serum and infection serum. The native TsSP proteins with 25–47 kDa in *T*. *spiralis* ML crude and ES proteins were recognized with anti-rTsSP serum (Fig 6B). Furthermore, the rTsSP was also recognized by immune serum from mice immunized with ML ES or crude antigens (Fig 6C). The results indicated that TsSP is one protein component from somatic and ES products of *T*. *spiralis* ML.

### RT-PCR assay of TsSP transcription at different stages

**Fig 7:**
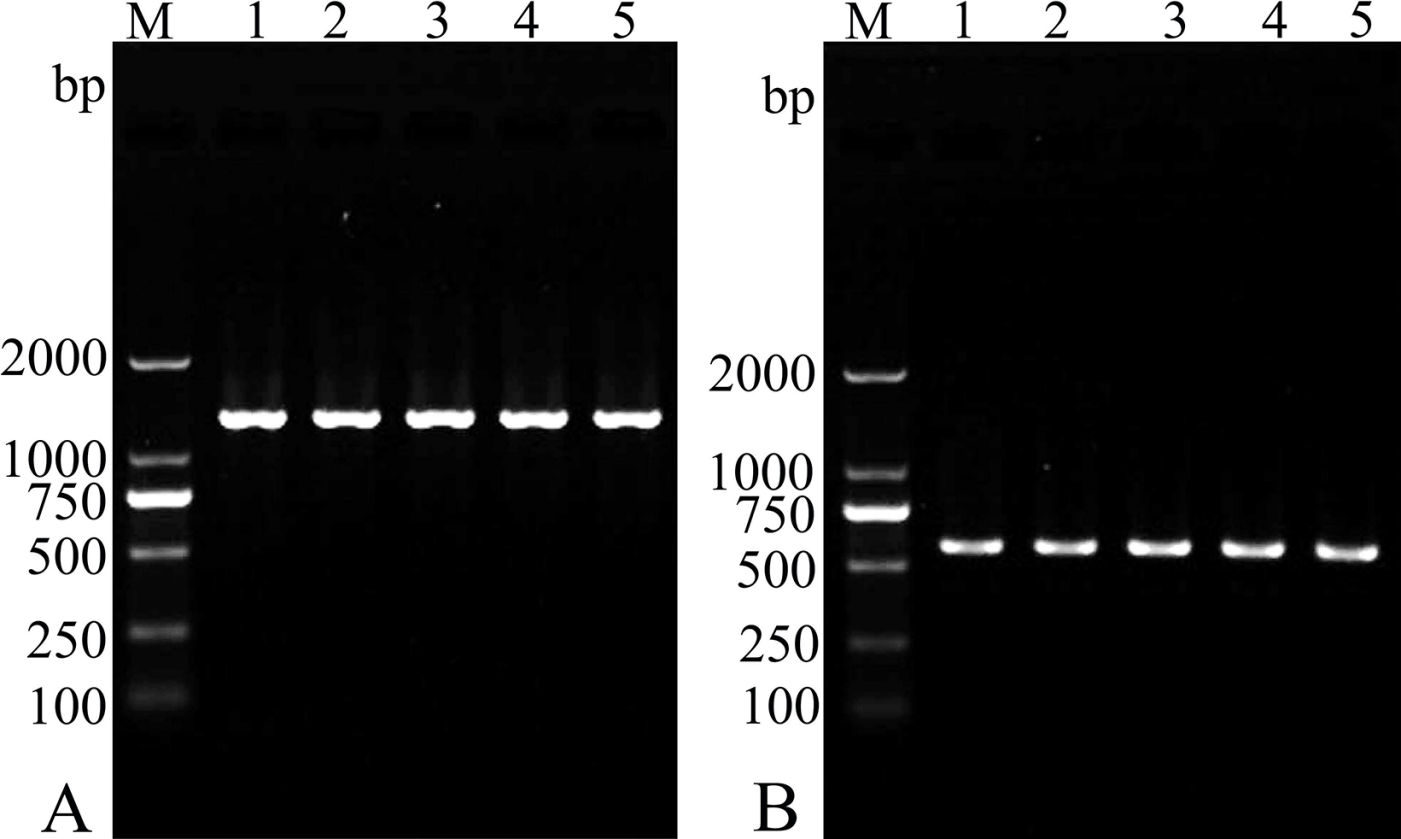
RT-PCR assay of TsSP transcription at different *T*. *spiralis* phases. RT-PCR assay of the mRNA transcription of TsSP (A) and GAPDH (B) at various *T*. *spiralis* stages. M: DNA marker; Lane 1: ML; Lane 2: IIL; Lane 3: AW at 3 dpi; Lane 4: AW at 6 dpi; Lane 4: NBL.

The TsSP transcription at different *T*. *spiralis* stages was assayed by RT-PCR assay and the transcription of GAPDH gene was used as an internal control. The TsSP mRNA transcript (1236 bp) was observed at all *T*. *spiralis* lifecycle stages (NBL, ML, IIL and AW). Moreover, the primers for the housekeeping gene (GAPDH) also produced the expected band (570 bp) in different developmental stages (Fig 7).

### ELISA and Western blot analysis of TsSP expression at various stages

**Fig 8:**
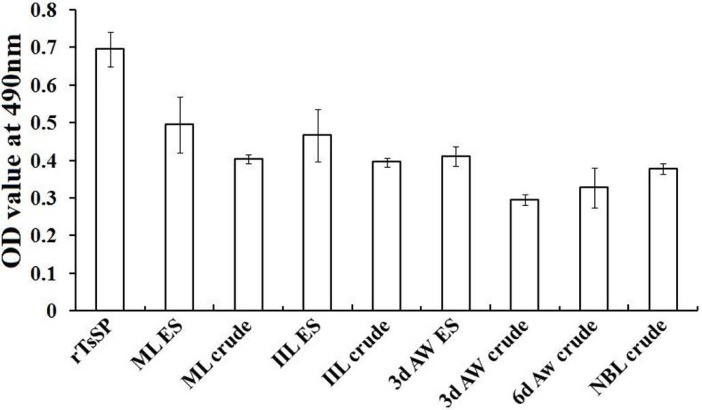
Anti-rTsSP IgG level of immunized mice determined by ELISA using antigens from various stages. OD values shown for each group (n = 13) were the arithmetic mean ± standard deviation (SD) of serum anti-rTsSP IgG levels.

**Fig 9:**
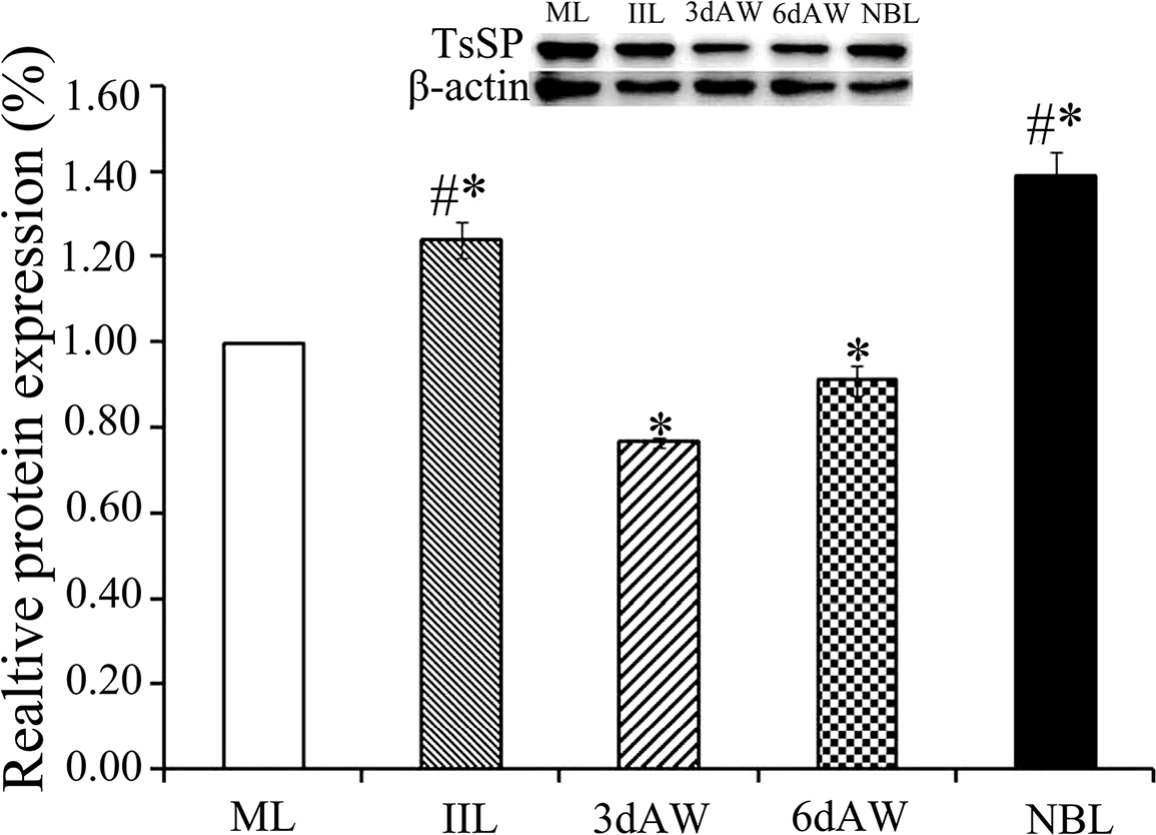
Western blot analyses of the TsSP expression levels at diverse *T*. *spiralis* phases. Expression levels of TsSP protein with 45.2kDa in crude antigens of diverse *T*. *spiralis* phase (AW, NBL, ML and IIL) were determined by Western blot with 1:100 dilutions of anti-rTsSP serum. The graph reveals the relative protein expression assayed with densitometry in 3 independent experiments. Asterisk (*) shows statistical differences (*P* < 0.05) relative to the ML. Pound sign (^#^) indicates remarkably statistical differences (*P* <0.01) relative the ML.

The results of ELISA revealed that the rTsSP and the native TsSP in crude and ES products of different stages (NBL, ML, IIL and AW) were identified by using anti-rTsSP serum (Fig 8). The results of Western blot analysis demonstrated that the native TsSP of 45.2 kDa in crude antigens of various stages were also probed with anti-rTsSP serum (Fig 9). These results further indicated that the TsSP was expressed at various developmental phases, and existed in both the somatic and ES proteins of the nematode. The TsSP expression level in IIL and NBL were obviously higher than those in the other three stages (ML, AW at 3 and 6 dpi) (*P* < 0.05).

### Immunolocalization of TsSP by IFT

**Fig 10:**
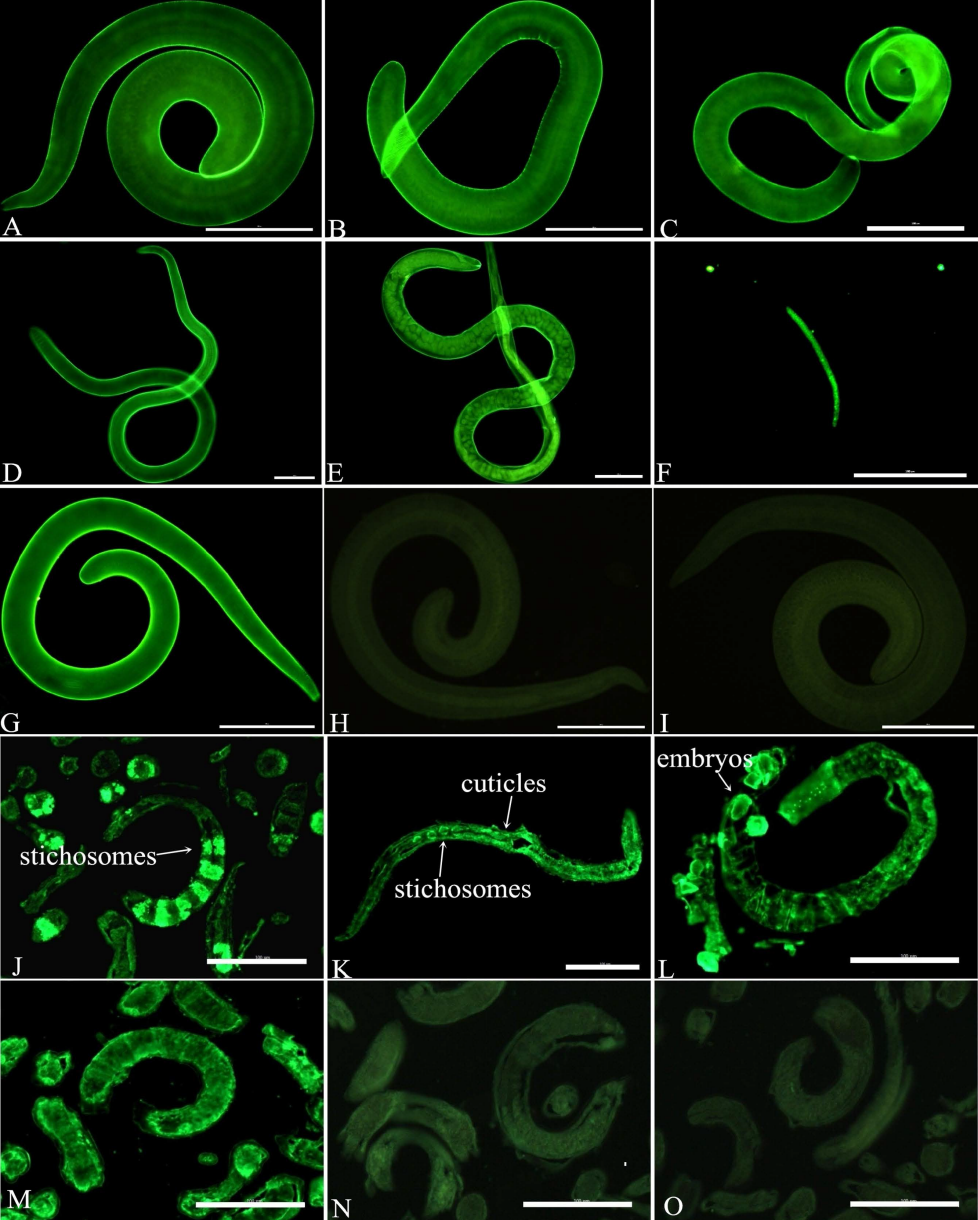
Immunolocalization of TsSP at different *T*. *spiralis* phases. A-I: IFT with intact worm of different *T*. *spiralis* phases probed by anti-rTsSP serum. The apparent immunostaining is observed on cuticles of ML (A), 6 hpi IIL (B), 24 hpi IIL (C), 3 dpi female adult (D), 6 dpi female adult (E), and NBL (F). The ML probed by infection serum (G) was utilized as a positive serum control; the ML incubated with normal mouse serum (H) and PBS (I) were applied to negative controls. When worm sections were incubated by anti-rTsSP serum, positive staining is seen in cuticles and stichosomes of ML (J), IIL (K) and female adult at 3 dpi (L). The ML probed by infection serum (M) was employed as a positive serum control; ML reveals no staining with normal mouse serum (N) and PBS (O) as a negative control. Scale-bars: 100 μm.

The IFT using intact parasite revealed that the immunostaining was found on cuticles of different stages (AW, NBL, ML and IIL) by using anti-rTsSP serum (Fig 10). While tissue sections of the nematode were incubated by anti-rTsSP serum, the staining was detected in cuticles and stichosomes of ML, IIL, AW and the embryos within uterus of female adult at 3 dpi.

### Detection of anti-*Trichinella* IgG in patients

**Fig 11:**
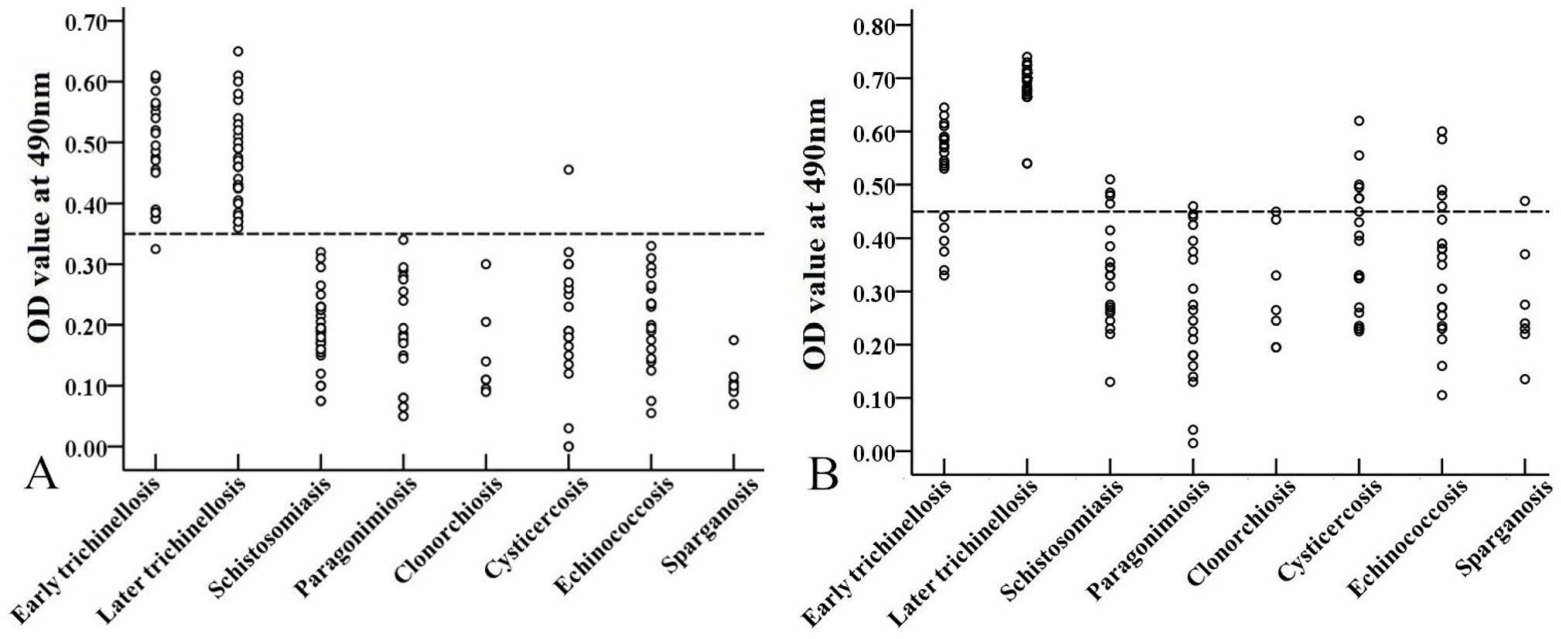
**Scatter plot of optical density values of rTsSP-ELISA (A) and ES-ELISA (B) for detection of anti-*Trichinella* IgG in serum samples of patients with trichinellosis and other parasitic diseases.** The cut-off value is represented by the dotted line.

The sensitivity of rTsSP-ELISA and ES-ELISA for detection of anti-*Trichinella* IgG in serum samples from trichinellosis patients was 98.11% (52/53) and 88.68% (47/53), respectively (χ^2^ = 2.910, *P* = 0.088). As the patients’ serum samples at 35 dpi were tested, the sensitivity of two antigens reached 100% (32/32). Nevertheless, while the patients’ samples at 19 dpi were examined, the sensitivity of the rTsSP was 95.24% (20/21), which was obviously higher than 71.43% (15/21) of ES antigens (χ^2^ = 4.286, *P* = 0.038) (Table 1). The specificity of the rTsSP and ES antigens was 99.53% (211/212) and 91.98% (195/212) (χ^2^ = 14.853, *P* = 0), when they were applied for detecting anti-*Trichinella* IgG in sera of patients with other parasitosis and healthy individuals. The cross-reaction of rTsSP with sera of patients with other parasitic diseases was not observed except for one serum sample from patients with cysticercosis (Fig 11).

**Table 1:** Detection of anti-*Trichinella* IgG antibodies in serum samples of patients with trichinellosis and other parasitic diseases by rTsSP-ELISA.

Sera of patients with	No. of serum samples	ELISA with rTsSP antigens		ELISA with ML ES antigens	
		OD value ($\bar{x}$ ± *S*)	No. of positive serum samples (%)	OD value ($\bar{x}$ ± *S*)	No. of positive serum samples (%)
Trichinellosis	53	0.47±0.09	52 (98.11)	0.62±0.11	47 (88.68)
Early trichinellosis*	21	0.48±0.08	20 (95.24)	0.52±0.10	15 (71.43)
Later trichinellosis^#^	32	0.47±0.08	32 (100)	0.69±0.04	32 (100)
Schistosomiasis	34	0.19±0.08	0 (0)	0.33±0.10	4 (11.76)
Paragonimiasis	20	0.18±0.09	0 (0)	0.26±0.14	1 (5.00)
Clonorchiasis	7	0.16±0.10	0 (0)	0.30±0.11	1 (14.29)
Cysticercosis	20	0.20±0.11	1 (5.00)	0.38±0.12	5 (25.00)
Echinococcosis	20	0.22±0.09	0 (0.00)	0.35±0.14	5 (25.00)
Sparganosis	7	0.11±0.03	0 (0)	0.27±0.10	1 (14.29)
Healthy persons	104	0.17±0.05	0 (0)	0.21±0.08	0 (0)

*Early trichinellosis: The sera of early patients with trichinellosis were collected at 19 days post infection.

^#^Later trichinellosis: The sera of later patients with trichinellosis were collected at 35 days post infection.

### Dynamics of serum anti-*Trichinella* IgG in experimentally infected mice

**Fig 12:**
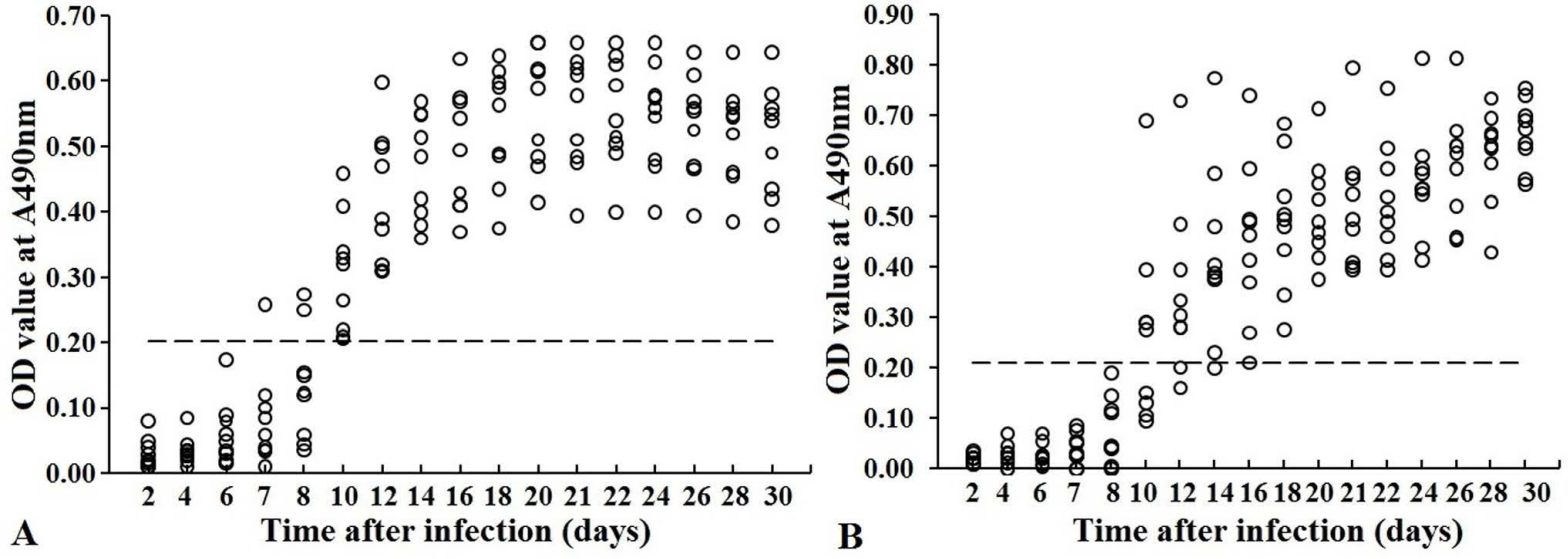
Kinetics of serum anti-*Trichinella* IgG in mice experimentally infected with 300 muscle larvae. Anti-*Trichinella* IgG was detected by rTsSP-ELISA (A) and ES-ELISA (B). The cut-off value is represented by the dotted line.

Serum anti-*Trichinella* IgG levels in infected mice at different time intervals post infection were measured by rTsSP-ELISA and ES-ELISA, respectively. Specific anti-*Trichinella* IgG was first detected by rTsSP-ELISA on 7 dpi and antibody positive rate reached 100% on 10 dpi (Fig 12A); when ES-ELISA was used, the specific antibody was first detected on 10 dpi and antibody detection reached 100% on 16 dpi (Fig 12B).

## Discussion

Previous studies showed there is an evident 2–3 week window of anti-*Trichinella* IgG negative after *Trichinella* infection, the antibody detection rate could not attain 100% till 1–3 months following *Trichinella* infection in humans [43,44]. The conventional ELISA with ML ES antigens lacks perfect sensitivity at the beginning of *Trichinella* infection, so improvements of diagnostic antigens would be of clinical value. In theory, detection of circulating antigens or DNA from *T*. *spiralis* live worms seems an ideal early diagnostic method for trichinellosis. But the levels of *Trichinella* circulating antigens in serum samples are usually lower and its detection rate in patients with clinical trichinellosis was usually only 30–50% [45,46]. Moreover, the persistence of *Trichinella* DNA is transient in blood circulation and the feces of infected hosts [23,47]. Therefore, determination of *Trichinella* circulating antigens or DNA has not been used for diagnosis of human trichinellosis. Up to now, determination of anti-*Trichinella* IgG is the most widely applied diagnostic method of trichinellosis, which is recommended by WHO and the International Commission on Trichinellosis (ICT) [10,48]. Therefore, it would be beneficial to identify antigens better able to diagnose recent *Trichinella* infection.

Serine protease (or serine proteinase) is a superfamily of widespread proteolytic enzymes in parasites, they exert an important part in physiological and pathological proceses during parasite infection [49]. The protease is related with the larval invasion, molting, digestion and fibrinolysis in parasitic nematodes [50,51]. Previous studies indicated that some secreted serine proteases were found in ES products from *T*. *spiralis* ML and AW, including serine protease TspSP-1 and trypsin-like 45 kDa antigen [52,53], and the enzymic activity of the native serine proteases in *T*. *spiralis* ML and AW ES proteins was also detected by biochemistry assay [54,55]. Our previous studies demonstrated that while the ML were activated into IIL and cocultivated with intestinal epithelial cells (IEC), the serine protease expression level in IIL stage was evidently increased as compared with ML stage [56,57], suggeting that the serine proteases might be involved in the larval invasion of host’s enteral mucosa. These serine proteases might be the target molecular antigens of the early host’s immune response, and they are possiblly used as the new diagnostic antigens for early trichinellosis [58].

The complete TsSP cDNA sequence was cloned and expressed in this study. The TsSP is attributed to the trypsin-like serine protease superfamily and has 90% identity with *T*. *nativa* which is another encapsulated *Trichinella* species [59]. After being purified, the rTsSP was strongly immunogenic and used for generating anti-rTsSP antibodies. Immunization of mice with the rTsSP elicited specific humoral immune response against rTsSP. The ELISA results revealed that the titer of specific anti-rTsSP IgG in immune serum was 1:10^5^. On Western blotting, the rTsSP protein was recognized with anti-rTsSP serum and mouse infection serum. As shown in Fig 6B, by using anti-rTsSP serum several native TsSP proteins was identified in *T*. *spiralis* ML crude and ES antigens. The TsSP might have different isoforms, or the protein was possibly processed by means of post-translational modifications/alternative splicing [11,60,61]. The process might be involved in the phosphorylation, methylation or acetylation of the TsSP after being translated, and they are possible important for the biological functions of the TsSP [38,62,63]. Additionally, it is also possible because the TsSP is a member of serine protease family, and they have the same functional domains.

The TsSP mRNA transcription was detected by RT-PCR at all *T*. *spiralis* developmental stage (AW, NBL, ML, IIL) (Fig 7). The TsSP expression was found by ELISA at various stage, but as shown in Fig 9, the TsSP expression level in IIL and NBL were obviously higher than those in the other three stages (ML, AW at 3–6 dpi) on Western blot anlysis. The IFT results demonstrated immunostaining was principally located in cuticle and stichosome of the nematode (Fig 10). Our results indicated that the TsSP was expressed at various *T*. *spiralis* phases and the TsSP was likely from the worm’s ES products. Previous studies showed another serine proteases (TspSP-1.2) was also expressed in *T*. *spiralis* different stages [38]. The results suggested that the TsSP is an essential protein and act a pivotal part in *T*. *spiralis* larval invasion and development. The the enzymatic activity and biological funtions of the rTsSP need to be studied in further experiments.

To investigate the potential use of rTsSP for serodiagnosis of human trichinellosis, rTsSP-ELISA method was establised and applied to assay anti-*Trichinella* IgG in trichinellosis patients’ serum samples, and the sensitivity was compared with those of ES-ELISA. The results revealed that the sensitivity of rTsSP-ELISA and ES-ELISA was 98.11% (52/53) and 88.68% (47/53), respectively (*P* > 0.05). Nevertheless, while the trichinellosis patients’ serum samples at 19 dpi were examined, the sensitivity (95.24%) of rTsSP was significantly higher than 71.43% (15/21) of ES antigens (*P* < 0.05), demostrating that the rTsSP protein was useful for the early diagosis of human trichinellosis. The specificity (99.53%) of the rTsSP was also superior to 91.98% of the ES antigens (*P* < 0.01). The cross-reaction of the rTsSP was seen only with one out of 20 serum samples of cysticercosis patients. The sensitivity and specificity of rTsSP are similar with that of recombinant *T*. *spiralis* 31 kDa protein [11]. The sensitivity of rTsSP for diagnosing early trichinellosis is comparative to those of ELISA using IIL or AW ES antigens, but the specificity of rTsSP-ELISA has an evident advantage over those of IIL and AW ES antigens [16,17]. Importantly, the anti-*Trichinella* IgG in 100% of the mice infected with 300 muscle larvae was detected by rTsSP-ELISA as soon as 10 dpi, but the ES-ELISA did not permit detection of 100% of infected mice before 16 dpi. The results suggested that the TsSP protein might be secreted by the nematode into the host’s peripheral blood circulation at the early infection stage and elicited an early specific anti-*Trichinella* antibody response continuing to the muscle stage [16]. Furthermore, our previous study has showed that the rTsSP could be recognized by early mouse infection sera at 8–10 dpi on Western blotting analysis [58]. Consequently, the rTsSP could be of value as potential novel antigen for the early diagnosis of *T*. *spiralis* infection in humans.

In summary, this study demonstrated that the TsSP was expressed at various *T*. *spiralis* developmental stages, it was likely from the worm’s ES products, and mainly located in cuticle and stichosome of this nematode. The rTsSP was strongly immunogenic. Sensitivity and specificity of rTsSP for detecting anti-*Trichinella* IgG antibodies are superior to the conventional ML ES antigens which are widely used at present. The rTsSP had the potential valuable as a new diagnositic antigen for early trichinellosis. But more serum samples from patients with trichinellosis and other nematode infection (ascariasis, trichuriasis, hookworm infection, filariasis, *etc*.) should be tested to further evaluate its sensitivity and specificity.
